# Limitations of Airway Dimension Measurement on Images Obtained Using Multi-Detector Row Computed Tomography

**DOI:** 10.1371/journal.pone.0076381

**Published:** 2013-10-08

**Authors:** Tsuyoshi Oguma, Toyohiro Hirai, Akio Niimi, Hisako Matsumoto, Shigeo Muro, Michio Shigematsu, Takashi Nishimura, Yoshiro Kubo, Michiaki Mishima

**Affiliations:** 1 Department of Respiratory Medicine, Graduate School of Medicine, Kyoto University, Kyoto, Japan; 2 Department of Medical Oncology and Immunology, Nagoya City University Graduate School of Medical Sciences, Nagoya, Japan; 3 Department of Respiratory Medicine, Sumitomo Hospital, Osaka, Japan; 4 Department of Respiratory Medicine, Kyoto-Katsura Hospital, Kyoto, Japan; 5 Department of Respirology, Kansai Electric Power Hospital, Osaka, Japan; University of Navarra, Spain

## Abstract

**Objectives:**

(a) To assess the effects of computed tomography (CT) scanners, scanning conditions, airway size, and phantom composition on airway dimension measurement and (b) to investigate the limitations of accurate quantitative assessment of small airways using CT images.

**Methods:**

An airway phantom, which was constructed using various types of material and with various tube sizes, was scanned using four CT scanner types under different conditions to calculate airway dimensions, luminal area (Ai), and the wall area percentage (WA%). To investigate the limitations of accurate airway dimension measurement, we then developed a second airway phantom with a thinner tube wall, and compared the clinical CT images of healthy subjects with the phantom images scanned using the same CT scanner. The study using clinical CT images was approved by the local ethics committee, and written informed consent was obtained from all subjects. Data were statistically analyzed using one-way ANOVA.

**Results:**

Errors noted in airway dimension measurement were greater in the tube of small inner radius made of material with a high CT density and on images reconstructed by body algorithm (p<0.001), and there was some variation in error among CT scanners under different fields of view. Airway wall thickness had the maximum effect on the accuracy of measurements with all CT scanners under all scanning conditions, and the magnitude of errors for WA% and Ai varied depending on wall thickness when airways of <1.0-mm wall thickness were measured.

**Conclusions:**

The parameters of airway dimensions measured were affected by airway size, reconstruction algorithm, composition of the airway phantom, and CT scanner types. In dimension measurement of small airways with wall thickness of <1.0 mm, the accuracy of measurement according to quantitative CT parameters can decrease as the walls become thinner.

## Introduction

Computed tomography (CT) is considered a useful technique for assessing airway dimensions and it is widely used for noninvasive in vivo structural evaluation of airway remodeling in asthma and chronic obstructive pulmonary disease (COPD) in clinical research. Developments in CT scanners and techniques of image analysis have contributed to the quantitative analysis of airways by CT density [1]–[3], as well as the visual assessment of airways. CT indices such as the ratio of airway wall area (WA) to total airway wall area (WA%) and luminal area (Ai) have been used for the quantitative analysis of airway thickening and narrowing. In COPD, the structural changes and narrowing of airways caused by chronic inflammation, in combination with emphysema, contribute to airflow limitation [4]. In addition, the small airways are key sites of obstruction [5]. Hence, structural assessment by CT has focused on the smaller and more distal airways. Some reports have described airway dimension measurement in small bronchi up to the 6^th^ or 10^th^ generation of airway branching [6]–[8], whereas other investigators have reported on the limitations of accurate CT measurement of small airways [9]. To validate the methods of airway dimension measurement, airway phantom models are widely used. However, in some reports airway dimension measurements have been performed outside the range validated by their phantoms [6]–[8], and the materials used for constructing airway phantoms have been varied–e.g., tubes made of polyethylene [3], acrylic resin [6], and silicone [7]. In these studies, they used different phantom materials, different CT scanners and different scanning conditions: hence, what had effects on errors in airway dimension measurement and what decided the limitations to accurate dimension measurement of small airways are not clear. In addition, to date, no report has examined the effects of phantom materials and their CT density on errors in airway dimension measurement using different CT scanners and scanning conditions.

Thus, the purpose of this study was to assess the effects of CT scanners, scanning conditions, airway size, and phantom construction on airway dimension measurement and then to investigate the limitations of accurate quantitative CT assessment of small airways.

## Materials and Methods

### Ethics Statement

The study using clinical CT images was approved by the ethics committee of Kyoto University (approval No. E-829), and written informed consent was obtained from all subjects.

### 1. The Effects of Scanning Conditions and CT Scanner Type on Errors in Airway Dimension Measurement: Phantom Study

#### Airway phantom

**Figure 1:**
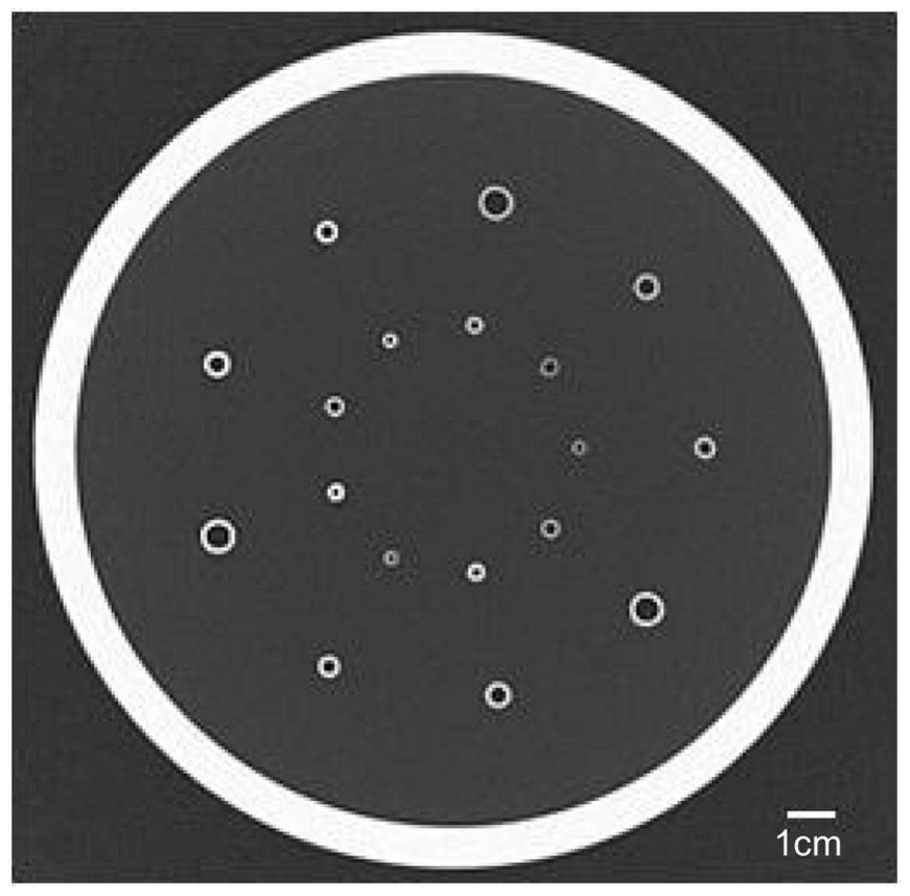
Axial slice computed tomography (CT) image of phantom A. The inner space of the cylindrical container is filled with successive layers of three materials: phenol resin, acrylic foam, and air. A total of 18 tubes (three materials×six sizes) were embedded through each layer (50 or 60 mm in length).

An airway phantom (phantom A: Kyoto Kagaku Co., Ltd., Kyoto, Japan) comprising various sets of different materials and tube sizes was used in this study. The tubes (5-cm long) were composed of three types of material [fluorocarbon polymers (physical density: 2.1 g/cm^3^), acrylic resin (1.2 g/cm^3^), and polyethylene (0.9 g/cm^3^)] that are embedded in three types of material mimicking lung parenchyma [phenol resin (0.32 g/cm^3^), acrylic foam (0.10 g/cm^3^), and air]. In addition, six sets differing in the inner radius and wall thickness were made for each different tube material type (Table 1). The tubes were measured by a digital caliper (accuracy of wall thickness ≤0.03 mm). All tubes were placed circularly in the same manner, regardless of material type, and were embedded in each of the three phantom lung parenchyma materials (Figure 1). Using this phantom, a total of 54 sets of tubes were analyzed, comprising combinations of three lung materials, three tube materials, and six tube sizes.

**Table 1:** Tube sizes in airway phantom A.

	Tube Number					
	1	2	3	4	5	6
Inner radius (mm)	3	2	1.5	1	1.5	1
Wall thickness (mm)	1	1	1	1	0.5	0.5

#### CT scans

Computed tomography scanning was performed in helical mode using four types of multi-detector row CT (MDCT) scanners with 64 detectors (Table 2). We used Aquilion 64 (Toshiba, Tokyo, Japan) as the primary unit, then examined the differences in results between this equipment and the other three CT scanners (Light Speed VCT, GE Healthcare UK, Buckinghamshire, UK; Brilliance 64, Philips, Eindhoven, Netherlands; and SOMATOM Definition, Siemens, Munich, Germany). CT scans were acquired from four scanners with various scanning conditions and reconstructions shown in Table 2. The phantom was placed on the table strictly perpendicular to the scan slices. To measure the tube sections at different oblique angles, the phantom was then placed obliquely to the scan slices at 30° intervals from 0° to 90° when scanned using Aquilion 64 with the scanning parameters of 120 mAs and 350-mm FOV.

**Table 2:** The four CT scanners used in assessments and their respective scanning data.

	CT Scanner			
	Aquilion 64	Light Speed VCT	Brilliance 64	SOMATOM Definition
kVp (kV)	120	120	120	120
Exposure (mAs)	120/AEC	120/60	120/60	120/60
FOV (mm)	350/200	350/200	350/200	350/200
Reconstruction algorithm	FC13/FC51/FC56	Standard/Lung	B/YA	B30/B70
Slice thickness and interval (mm)	1/0.5	0.625	0.67	0.6

kVp, kilovolts peak; FOV, field of view; AEC, automatic exposure control (actual range in this study: 25–30 mAs).

#### Airway dimension measurement

**Figure 2:**
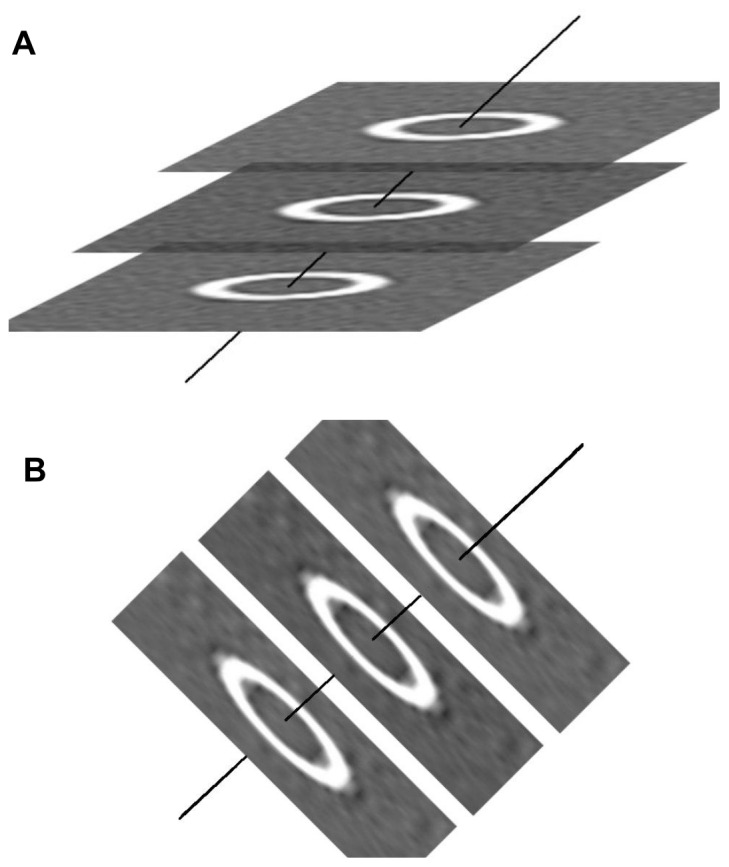
Schema describing the method of airway dimension measurement. A: Using sequential CT slices which included a section of the target tube, the center (solid) line of the tube was calculated by linking the center points of sections on each slice. B: Images were constructed perpendicular to the center line.

Airway measurements were made using software described previously, with modifications [3]. This software analyzed the dimensions of airways and tubes as follows: first, the section in which the tube had the smallest area and greatest circularity was selected as the provisional section for each of the seven planes: i.e., horizontal, coronal, sagittal, and the planes passing through the middle of these standard three planes. Second, the center line of the tube was calculated by linking the center points of the luminal areas of the provisional section with the adjacent front and rear sections (Figure 2A). Third, the wall and lumen of the tube were reconstructed three-dimensionally along the center line (Figure 2B). Fourth, slice images perpendicular to the center line were reconstructed using trilinear interpolation, and on these images Ai, WA, and wall thickness (WT) were calculated using the full-width half-maximum (FWHM) method, as reported previously [3]. WA% was defined as WA/(Ai+WA)×100. All these steps were performed using images with a 4×magnification, and the middle two-thirds of the tubes in phantom (3.3 cm long, about 190 slices) were analyzed. Finally, mean values of Ai, WA%, and WT were calculated.

#### Comparison between actual values and CT measurements

We assessed the errors in CT measurement as a percentage of the actual values using the following formula:
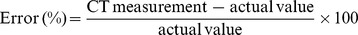



### 2. Limitations of Airway Dimension Measurement Using Clinical CT Images and a Second Airway Phantom

To apply the phantom study for clinical CT images and investigate how distal generation of airway branching can be measured accurately, chest CT images in 10 healthy adults (mean age: 62.0 years, range: 38–80 years; male/female: 2/8) were used to analyze airway dimensions of the right posterior basal bronchus and more distal bronchi (3^rd^–6^th^ generation) by the same method as in the phantom study. All subjects visited Kyoto University Hospital for further examination of chest X-ray abnormalities and underwent CT scanning with Aquilion 64 (350-mm FOV and lung algorithm FC56). No contrast media were used. Subjects had no respiratory symptoms, no history of respiratory disease, and no abnormal findings on spirometry or chest CT.

According to the results obtained using airway phantom A, we developed a second, thin-walled airway phantom (phantom B) to assess the lower limitations of airway dimension measurement. In phantom B, six acrylic resin tubes (inner radius: 1.5 mm) with varying wall thickness (1.0, 0.9, 0.8, 0.7, 0.6, 0.5 mm) were placed circularly and embedded in air. This phantom was then scanned using Aquilion 64 (350-mm FOV and lung algorithm FC56).

### Statistical Analysis

Data were statistically analyzed using one-way ANOVA in differences between materials, tube sizes, scanners, and scanning conditions with JMP 6.0.3 software (SAS Campus Drive, Cary, NC, USA). Graphs were displayed as average with standard deviation (SD).

## Results

### Effect of Scanning Conditions on Airway Dimension Measurements

**Figure 3:**
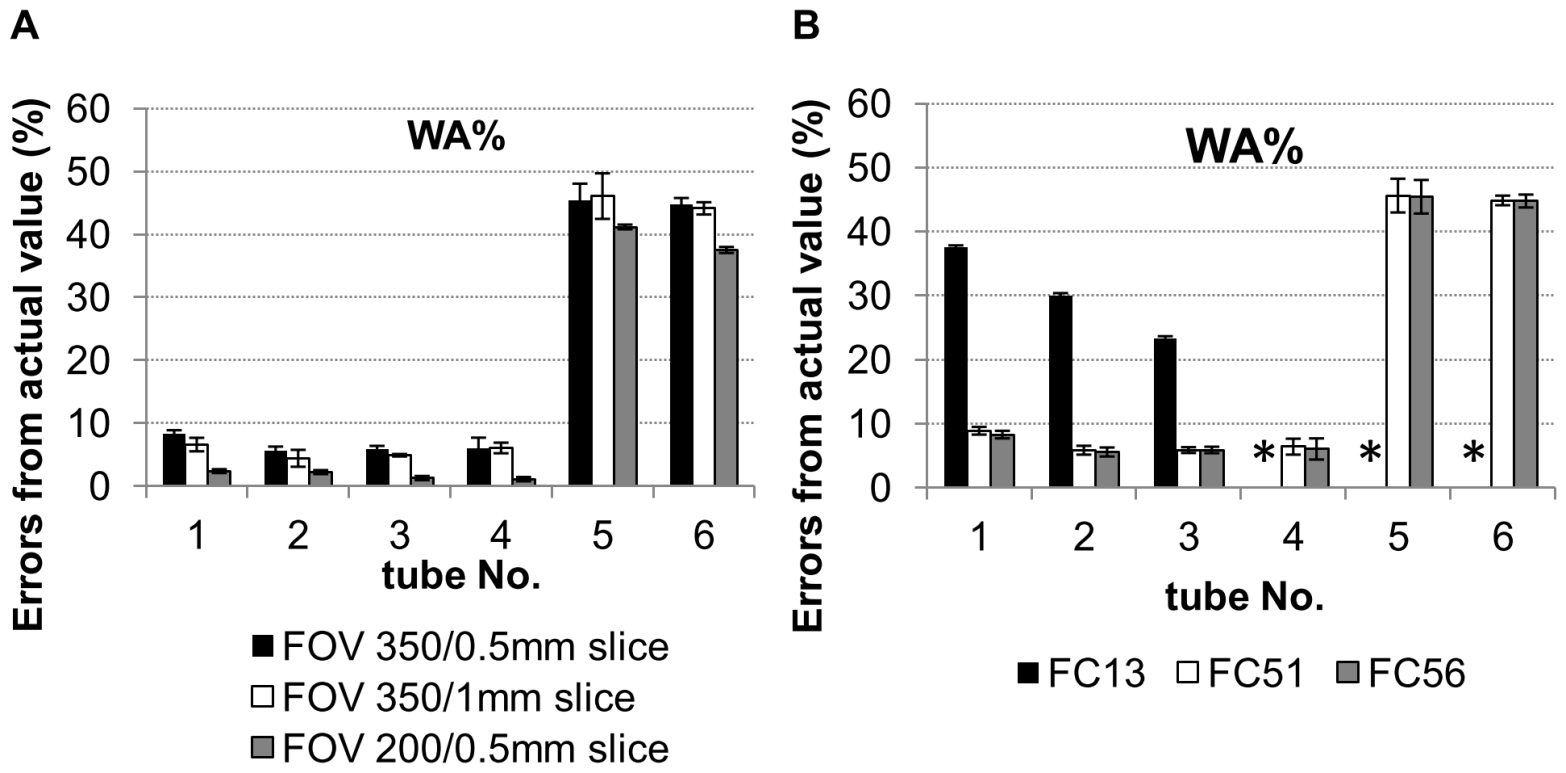
Effects of scanning conditions on errors of airway dimension measurement. A: Effects of field of view (FOV) and slice thickness on errors for wall area percentage (WA%) in acrylic resin tubes surrounded by acrylic foam that were scanned using Aquilion 64 (120 mAs and lung algorithm FC56). B: Effects of the reconstruction algorithm on the errors of WA% in acrylic resin tubes surrounded by acrylic foam that were scanned using Aquilion 64 (120 mAs, 0.5-mm slice thickness, 350-mm FOV). FC13: body algorithm, FC51: lung algorithm, FC56: lung algorithm (FC51) with beam-hardening correction. *: failure to measure. The error of airway dimensions was defined as follows: Error (%) = (CT measurement − actual value)/actual value×100.

Figure 3A shows the errors recorded using Aquilion 64 from actual values of WA% on images under varying FOVs and slice thickness. Although the images scanned under lower FOVs were associated with smaller error values, errors in tubes #5 and #6 with 0.5-mm wall thickness were >32% for all combinations of FOV and slice thickness. The differences in errors between slice thicknesses were less than those among FOVs. These results were similar to the errors recorded for Ai.

Variations in radiation exposure (measured in mAs) had very little effect on measured values. Maximum differences in error for WA% between two exposures were 0.26%, 0.95%, 0.56%, and 0.72% for Aquilion 64, Light Speed VCT, Brilliance 64, and SOMATOM Definition, respectively.

Figure 3B shows a comparison of error according to WA% for each tube using the three different reconstruction algorithms [body algorithm (FC13), lung algorithm (FC51), and lung algorithm with beam-hardening correction (FC56)] using Aquilion 64. Errors were significantly greater on images reconstructed by the body algorithm than the lung algorithm (p<0.001 in tubes #1 to #3). Using the three other CT scanners, errors were also larger on images reconstructed by the body algorithm than the lung algorithm.

### Effect of Phantom Materials on Measurement of Tube Dimensions

**Figure 4:**
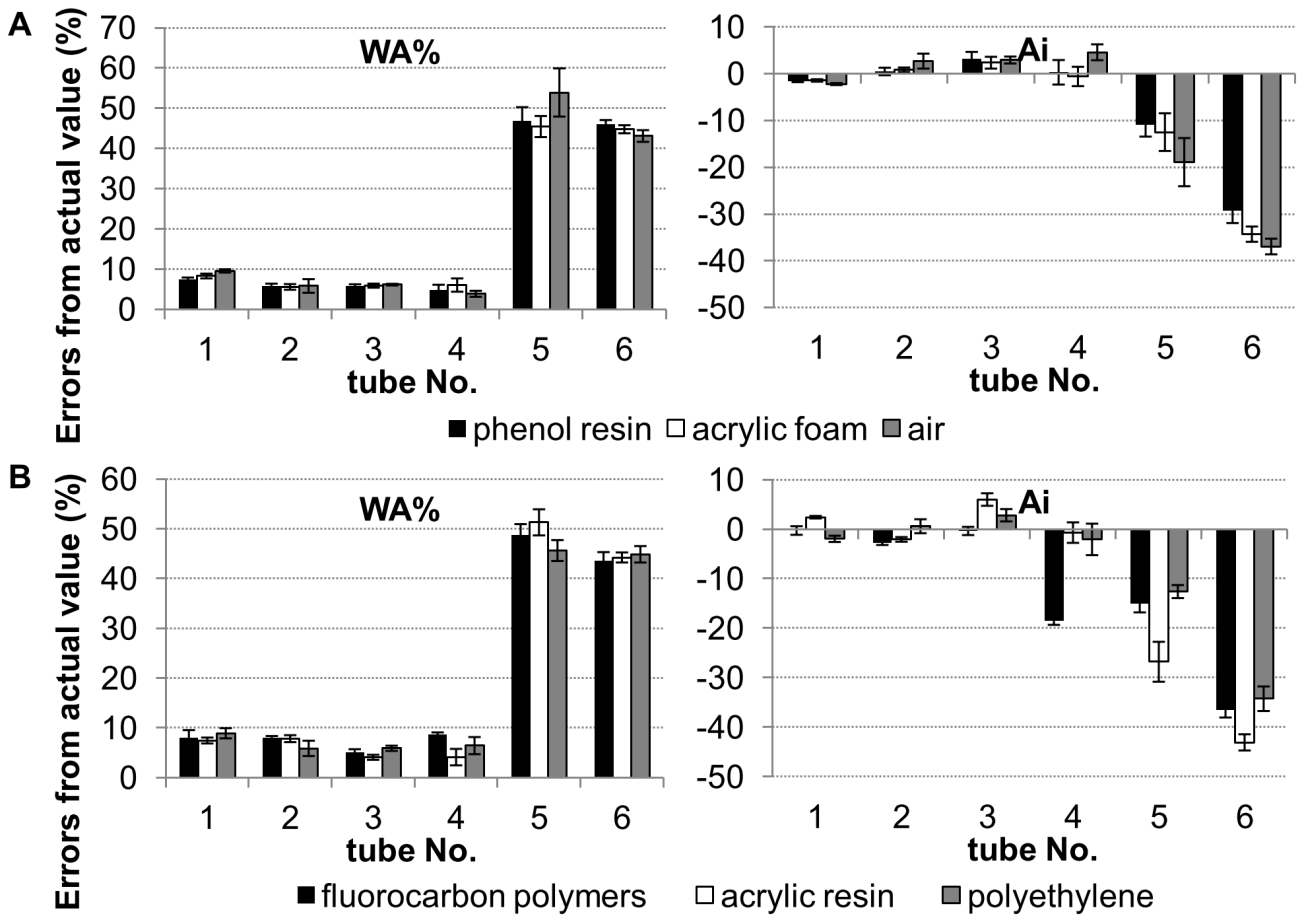
Effects of phantom composition on errors of airway dimension measurement. Percentage error of wall area (WA%) and luminal area (Ai) for the phantom scanned using Aquilion 64 (120 mAs, 0.5-mm slice thickness, 350-mm FOV, lung reconstruction algorithm FC56). A: Comparison of errors of WA% and Ai for acrylic resin tubes among materials simulating lung parenchyma, phenol resin (0.32 g/cm^3^), acrylic foam (0.10 g/cm^3^), and air. B: Comparison of errors for WA% and Ai among tube materials, fluorocarbon polymers (2.1 g/cm^3^), acrylic resin (1.2 g/cm^3^), and polyethylene (0.9 g/cm^3^) embedded in acrylic foam.

The effects of differences in phantom materials on measurement of tube dimensions are shown in Figure 4. The mean values of CT density in the phantom materials mimicking lung parenchyma and the mean values of maximum CT density in the tube walls on the images scanned using four scanners (120 mAs, 350-mm FOV, and lung reconstruction algorithm) are shown in Table 3. On images scanned using Aquilion 64 (120 mAs, 0.5-mm collimation, 0.5-mm slice thickness, 350-mm FOV, and lung reconstruction algorithm FC56), the errors in WA% and Ai for acrylic resin tubes enclosed by the three different materials used in the lung phantom are shown in Figure 4A. The effect of differences in materials used for the simulated lungs was quite small (<5.2% in tubes #1 to #4). Figure 4B shows errors for WA% and Ai in tubes made of the three different materials surrounded by acrylic foam. The absolute value of error of measured Ai in the tube made of fluorocarbon polymers with 1.0-mm inner radius and 1.0-mm wall thickness (tube #4) was much greater than that in the tubes made of other materials (p<0.001). Thus, the minimum limit of tube size that could be measured with small error was greater in fluorocarbon polymer tubes than in tubes made of polyethylene or acrylic resin.

**Table 3:** The mean values of CT density (HU) in the phantom materials mimicking lung parenchyma and the mean values of maximum CT density in the tube walls.

	CT Scanner			
	Aquilion 64	Light Speed VCT	Brilliance 64	SOMATOM Definition
phenol resin	−653.1 (39.3)	−666.7 (24.2)	−671.4 (27.7)	−672.5 (37.6)
acrylic foam	−930 (25.7)	−923.4 (16.0)	−923.4 (18.1)	−918.7 (26.2)
air	−1014 (22.3)	−999.83 (12.3)	−999.8 (14.5)	−999.0 (20.7)
fluorocarbon polymers	1163.4 (134.4)	1370.3 (152.0)	543.6 (73.3)	1326.2 (166.6)
acrylic resin	209 (80.6)	348.5 (87.7)	−108.6 (34.3)	285.2 (93.2)
polyethylene	−31.4 (61.1)	39.5 (63.8)	−295.0 (30.8)	3.0 (76.6)

Average of measured values (SD).

### Effect of Phantom Angles on Measurement of Tube Dimensions

The results described above were similar even when the phantom was placed obliquely to the scanning section at various angles (0°–90°). Absolute values of error in tubes #5 and #6, both with wall thickness of 0.5 mm, were >38% at all angles. Maximum differences in errors between phantom angles were small (<7%) (Table 4). These results were similar in images with 1.0-mm slice thickness, and maximum differences in errors between phantom angles were <10%.

**Table 4:** Effects of phantom angles on errors of airway dimension measurement.

angle	Tube Number					
	1	2	3	4	5	6
0°	8.27(0.59)	5.56(0.70)	5.87(0.5)	6.02(1.65)	45.43(2.62)	44.76(1.01)
30°	7.61(0.92)	4.11(1.43)	5.16(1.13)	3.88(0.57)	46.77(0.81)	40.93(2.28)
60°	6.68(0.58)	5.52(0.98)	4.64(0.46)	5.01(0.90)	46.34(1.69)	40.21(1.40)
90°	6.73(0.94)	7.31(0.08)	3.89(0.02)	5.93(1.18)	49.45(1.50)	38.39(1.61)

Percent errors from actual value (SD).

Errors of WA% in acrylic resin tubes embedded in acrylic foam at various angles using Aquilion 64 (120 mAs, 0.5-mm slice thickness, 350-mm FOV, lung reconstruction algorithm FC56).

### Difference between CT Scanners

**Figure 5:**
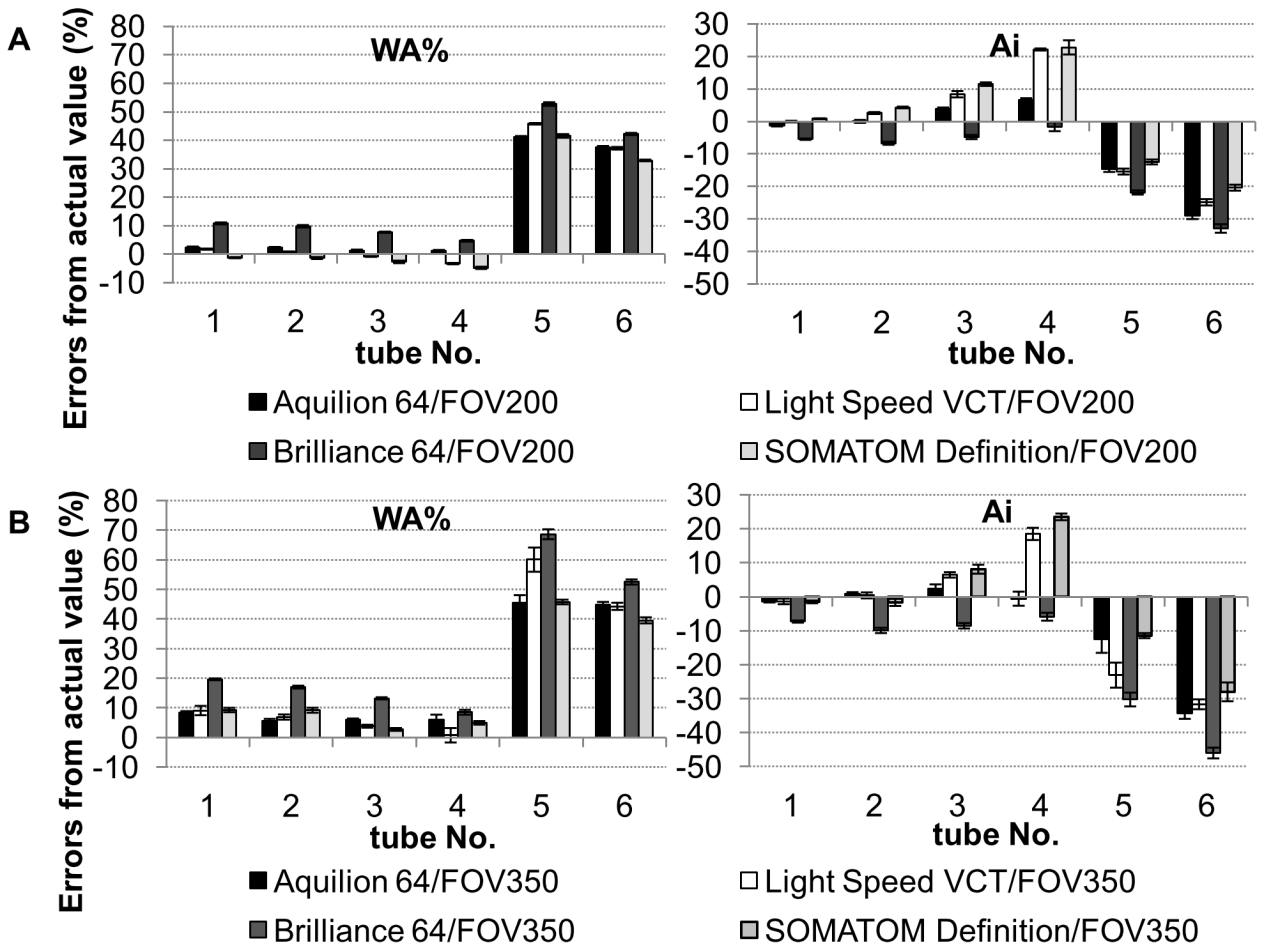
Effects of CT scanner and FOV on errors of airway dimension measurement. Comparison of errors WA% and luminal area (Ai) in acrylic resin tubes embedded in acrylic foam among four CT scanners under varying FOV (A: 200 mm, B: 350 mm). The images were reconstructed by the lung algorithm. The definition of error is shown in the legend to Figure 3.

Figure 5 shows a comparison of errors for WA% and Ai among the four CT scanners under two different FOVs. In all CT scanners under both FOVs, the absolute values of errors for WA% and Ai in tubes of 0.5-mm thickness were >33% and 11%, respectively. However, there were certain differences in errors in tubes #1 to #4 among CT scanners under different FOVs. The errors for WA% in tubes #1 to #3 using Brilliance 64 under 350-mm FOV were greater than those using other scanners (p<0.001), and these errors using Brilliance 64 improved when scanning was carried out under 200-mm FOV. On the other hand, errors for Ai in one tube (#4) with 1.0-mm inner radius and 1.0-mm wall thickness using Light Speed VCT and SOMATOM Definition were greater (>18%), and these did not improve even under a smaller FOVs.

### Limitations of Airway Dimension Measurement Using Clinical Images and Airway Phantom B

**Figure 6:**
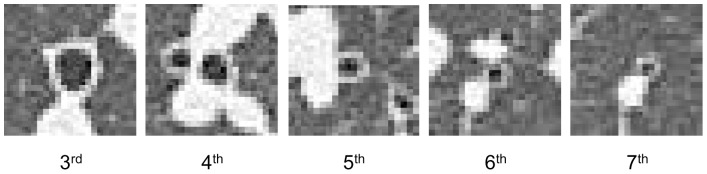
Examples of airways measured at different generations. The representative images of right posterior basal bronchi (3^rd^ generation) and more distal bronchi of a healthy control on Aquilion 64 (Auto Exposure Control, 0.5-mm slice thickness, 350-mm FOV, lung reconstruction algorithm FC56). At the 6^th^ to 7^th^ generation, the thickness of the bronchus wall had equal or less than pixels size.

Table 5 shows the dimension measurement of the airways of the right posterior basal bronchus (3^rd^ generation) and more distal bronchi. Although the average inner radius and WT of the 6^th^ bronchus were 1.44 mm and 1.06 mm, respectively, some subjects showed WT of <1.0 mm in the 5^th^ and more distal bronchi (Figure 6).

**Table 5:** Dimension measurement of the airways of the right basal bronchus (by generation) in healthy subjects.

	3^rd^	4^th^	5^th^	6^th^
Inner radius (mm)	2.50 (1.73–3.26)	2.27 (1.28–3.10)	1.79 (0.89–2.38)	1.44 (0.84–2.27)
Wall thickness (mm)	1.29 (1.12–1.48)	1.23 (1.01–1.57)	1.12 (0.98–1.30)	1.06 (0.93–1.31)

Average (range).

**Figure 7:**
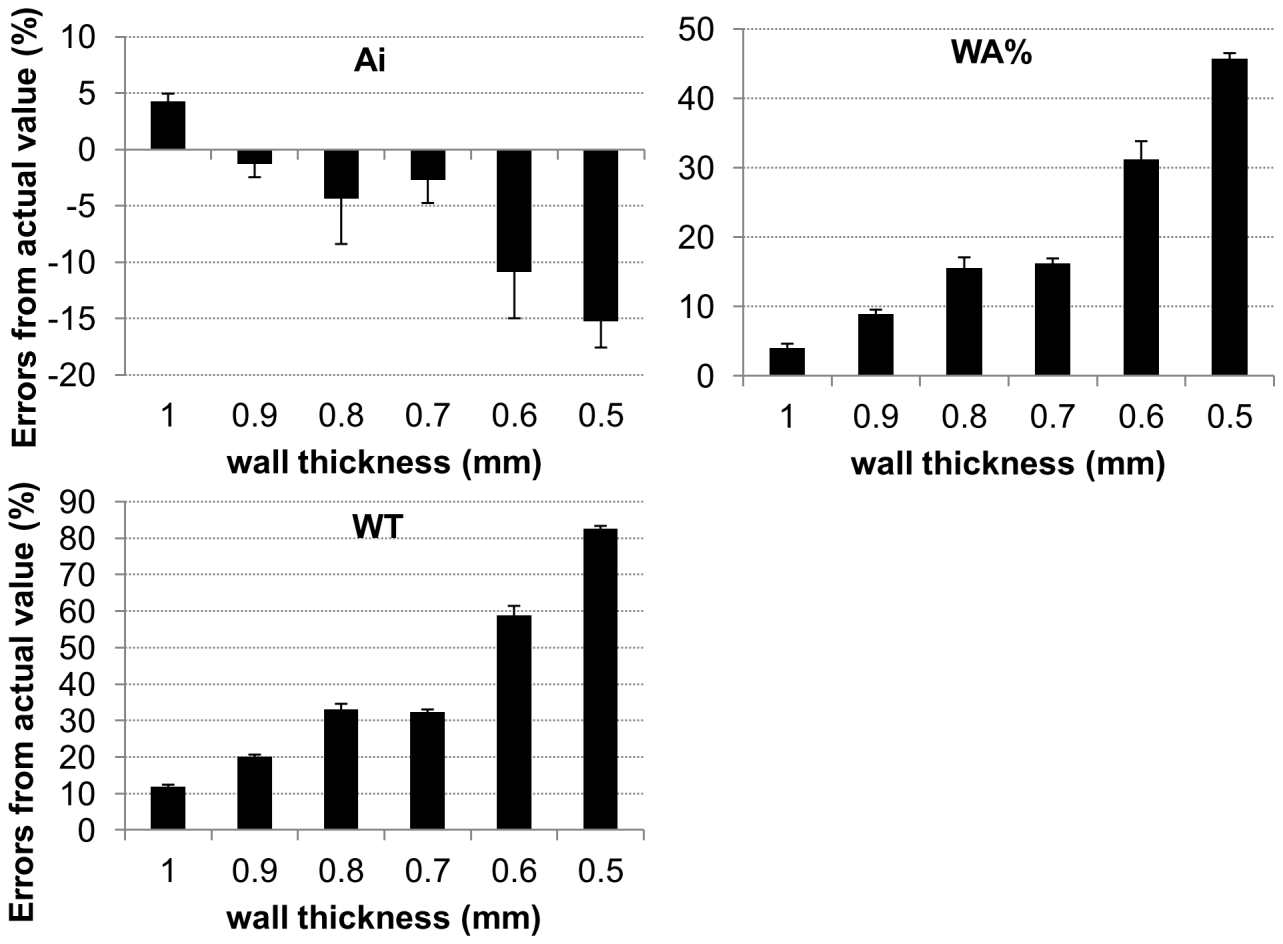
Effects of wall thickness on errors of airway dimension measurement. Comparison of errors of Ai, WA%, and wall thickness (WT) for various wall thickness using airway phantom B scanned by Aquilion 64 (120 mAs, 0.5-mm slice thickness, 350-mm FOV, and lung reconstruction algorithm FC56).

Figure 7 and Table 6 show a comparison of errors for Ai, WA%, and WT among tubes of varying wall thickness in the images of airway phantom B scanned using Aquilion 64. The errors for WA% and WT increased with thinness of airway wall, whereas the errors for Ai were <5% in tubes of ≥0.7-mm wall thickness.

**Table 6:** Effects of wall thickness on airway dimension measurement.

	Actual wall thickness (mm)					
	1	0.9	0.8	0.7	0.6	0.5
Ai (mm^2^)	7.37(0.05)	6.97(0.08)	6.76(0.29)	6.87(0.15)	6.30(0.29)	5.99(0.16)
WA% (%)	66.60(0.38)	66.41(0.35)	66.42(0.87)	62.18(0.40)	64.27(1.29)	63.76(0.35)
WT (mm)	1.12(0.01)	1.08(0.01)	1.06(0.01)	0.93(0.01)	0.95(0.02)	0.91(0.00)

Average of measured values (SD).

Measured values of Ai, WA%, and wall thickness (WT) for various wall thickness using airway phantom B (actual luminal area: 7.07 mm^2^) scanned by Aquilion 64 (120 mAs, 0.5-mm slice thickness, 350-mm FOV, and lung reconstruction algorithm FC56).

## Discussion

This study showed two main findings with regard to airway dimension measurement by CT imaging. First, the error of measurement varies with regard to CT scanner, reconstruction algorithm, and airway phantom construction. This suggests that in airway dimension measurement using clinical CT imaging, validation using the same scanner and scanning conditions is necessary, and materials of similar CT density to that of bronchial wall should be used for the airway phantom in validation studies. Second, errors in the widely used quantitative CT parameters, WA% and luminal area, could depend on WT, particularly when distal airways of <1.0-mm WT are measured.

To investigate the pathophysiology of obstructive lung disease, the assessment of airway remodeling in the smaller and more distal airways using MDCT was considered following the development of CT scanners and techniques of image analysis. Because CT images have certain limitations with regard to spatial resolution, it is important to be aware of the errors and limitations of airway dimension measurement. Thus, phantom studies have been widely used for the validation of methods. However, several reports using phantoms have presented findings for small and distal airway dimensions that were outside the range validated by their particular study [6]–[8]. Although Hasegawa et al. [6] showed that the average WT in the 6^th^ branch was 0.9 mm, wall thickness of the phantom tubes used in that validation study was 1.0 mm. Montaudon et al. [7], [8] reported that WT of the 10^th^ branch measured <0.2 mm, but the airway phantom in their validation study had wall thickness of ≥0.86 mm. Moreover, the phantom materials used for validation varied with the studies. For quantitative analysis using CT images, it is appropriate that the CT density of the phantom material simulates the density of the actual airway wall (0–400 HU) and surrounding lung parenchyma (−900 to −800 HU). However, to our knowledge, no reports have shown how phantom materials and their CT density affect airway dimension measurement. In the present study, the effects of lung phantom composition were limited. On the other hand, with regard to airway phantom composition, the errors for Ai were least in acrylic resin tubes. A small tube (1.0-mm inner radius) constructed from fluorocarbon polymers showed the largest error for Ai compared with those of other materials. Materials such as fluorocarbon polymers, which have a higher CT density than the human bronchial wall, may not be suitable for use in the airway phantom.

Next, using four CT scanner types, we investigated the effects of scanning conditions and reconstruction algorithm on the error of airway dimensions. The effects of radiation exposure and slice thickness were found to be very small in ranges used to assess airway dimensions. Robinson et al. [10] also reported that radiation dose had no effect on measurement error. Our results showed that, for all CT scanners used, the reconstruction function for lung images was correct for airway dimension measurement, whereas that for body images was not. Saba et al. [11] reported that mean error decreased as image sharpness increased when using the Imatron electron beam CT scanner, and Kim et al. [12] reported similar results using the Siemens Sensation 16 CT scanner when they analyzed images obtained using the FWHM method. Regarding the effect of FOV, Saba et al. [11] and Kim et al. [12] reported a similar error for all FOVs studied, whereas Takahashi et al. [13] reported that FOV had an inﬂuence on airway dimension measurement especially for the tubes of 1.0-mm wall thickness when using Light Speed VCT. In the present study, we found variation in the error of airway dimension measurement and in the effect of FOV among CT scanners. These results suggest that it is important to validate the characteristics of the method employed, including software and hardware, before obtaining clinical images.

The factor having the greatest effect on airway dimension measurement in the present study was wall thickness. Tubes of 0.5-mm wall thickness could not be measured with sufficient accuracy with any scanner under any scanning parameters, even when the inner radius (1.5 mm) was greater than the minimum limit of 1.0-mm wall thickness. This result can be explained by the fact that the dimension of 0.5 mm is close to the size of the detector and pixel dimensions that are responsible for resolution on clinical CT images. From our study using airway phantom B with thinner walls, the luminal area of tubes of ≥0.7-mm wall thickness could be accurately measured with an error <5%, whereas the error for WA% and WT increased with thinness of airway wall in tubes of <1.0-mm wall thickness. In practical use, when 1-mm wall thickness (error ∼10%) is set to be the limitation for accurate measurement, measured WT values less than 1.12 mm is not accurate in the present study (Table 6 and Figure 7). This means that some measured values in bronchial branching of the 5^th^ generation or more were not accurate in dimension measurement of the airways of the right basal bronchus using clinical images of healthy subjects (Table 5). The present study suggests that the quantitative CT parameters WA% and Ai may be associated with greater error depending on the thinness of the airway wall, especially when small and distal airways of ≤1.0-mm WT are measured. For example, the differences in WA% between healthy controls and patients with asthma were reported to be 5–10% [14], and thus, errors more than 10% can be too large to detect changes in diseases accurately. This means that there may be severe limitations to assess peripheral small airways directly using CT images.

There are some limitations to this study. First, we were unable to investigate the effects of different algorithms on errors in airway dimension measurement. Several alternative methods, such as the maximum-likelihood algorithm [15], a method of ellipse fitting to the airway lumen and wall [11], a score-guided erosion algorithm [16], and an integral-based method [17], have been reported previously. When using different algorithms for airway analysis, the effects on measurement accuracy may be different. However, the FWHM principle used in this study is the most widely used method, and there is no clear indication that any one algorithm provides more useful data than another [9]. Moreover, also in those reports, when wall thickness was <1.01–1.16 mm, measurement errors were >10% [11], [15]–[17], as shown in the present study using the FWHM principle. This may suggest that small airway measurement using any algorithms for the measurement of distance close to spatial resolution of CT images can have larger errors. A further limitation is that accuracy is not guaranteed in phantom airway dimension measurement. The inner and outer contours of actual airways are not always completely circular and their walls are not homogeneous with regard to physical density. However, to validate this method of analysis, a phantom study is required to define the errors of measurements, and it is widely used for such validation.

## Conclusions

In conclusion, the parameters of airway dimensions measured using CT images were affected by airway size, reconstruction algorithm, composition of the airway phantom, and CT scanner types. In dimension measurement of small airways with wall thickness of <1.0 mm, the accuracy of measurement according to quantitative CT parameters can decrease as the walls become thinner.
